# Physical analysis of the shielding capacity for a lightweight apron designed for shielding low intensity scattering X-rays

**DOI:** 10.1038/srep27721

**Published:** 2016-07-27

**Authors:** Seon Chil Kim, Jeong Ryeol Choi, Byeong Kyou Jeon

**Affiliations:** 1 Department of Biomedical Engineering, School of Medicine, Keimyung University, Dalgubeol-daero 1095, Daegu 42601, Republic of Korea; 2 Department of Radiologic Technology, Daegu Health College, Yeongsong-ro 15, Buk-gu, Daegu 41453, Republic of Korea

## Abstract

The purpose of this paper is to develop a lightweight apron that will be used for shielding low intensity radiation in medical imaging radiography room and to apply it to a custom-made effective shielding. The quality of existing aprons made for protecting our bodies from direct radiation are improved so that they are suitable for scattered X-rays. Textiles that prevent bodies from radiation are made by combining barium sulfate and liquid silicon. These materials have the function of shielding radiation in a manner like lead. Three kinds of textiles are produced. The thicknesses of each textile are 0.15 mm, 0.21 mm, and 0.29 mm and the corresponding lead equivalents are 0.039 mmPb, 0.095 mmPb, 0.22 mmPb for each. The rate of shielding space scattering rays are 80% from the distance of 0.5 m, 86% from 1.0 m, and 97% from 1.5 m. If we intend to approach with the purpose of shielding scattering X-rays and low intensity radiations, it is possible to reduce the weight of the apron to be 1/5 compared to that of the existing lead aprons whose weight is typically more than 4 kg. We confirm, therefore, that it is possible to produce lightweight aprons that are used for the purpose of shielding low dose radiations.

For the case of a general shooting using medical rays, there is a space dose, i.e., scattering X-rays, inside the photographing room. Due to this, there is the possibility of exposing low dose medical rays onto medical workers and patients. To prevent this, one must wear a special apron that shields medical rays. Typically, the weight of such an apron is 3.25 kg for the case that it is made of 0.25 mmPb, and 4.95 kg for 0.50 mmPb. The aforementioned weight would prove to be inconveniently heavy on the medical workers, thus making it difficult to efficiently move around to perform their required responsibilities^1^,^2^.

Recently, much effort has been paid to reducing the weight of the apron which, in essence, is designed to block as much as possible harmful medical rays from the user. As a radical resolution for this problem, there have been efforts for discovering alternative materials that have, at least, the same shielding capacity, manufacturing quality, and economical efficiency as those of lead. For candidate materials to be used, one could possibly implement a compound of tungsten, bismuth, barium, boron, and tin^3^.

Although there has been extensive research in the field of shielding direct rays when using radiation at medical institutes, the kinds of materials that can be used for shielding low dose radiation are still rare. In general, low intensity radiation means the radiation whose intensity is below 100 mSv. There is no direct evidence at present that cancers can be generated by such rays, but most medical rays belong to the low intensity radiation; hence, the dose of these rays are 20 to 30 times larger than the annual natural ray which is about 2.4~3.0 mSv^4^. In general, it has been thought that the workers who deal with medical rays in the photographing room are actively exposed to low intensity radiations^5^,^6^,^7^,^8^,^9^,^10^.

Thus, although it is desirable that one wears an apron when entering an area at the medical institute where radiations are generated, an effective shielding system with the standard wearing of an apron is necessary for the area of low dose. If one always wears a light apron or a working suit that is made using radiation dose reduction fiber in order to block rays at the work place, the danger of being exposed to the low intensity rays will be reduced significantly. Hence, if we consider that the main material of the typical existing aprons is the massive lead, the shielding products that are made of light materials without lead should exhibit the similar properties of ordinary fibers so that an apron-wearer could feel the sensations of improved lightness and softness. In this research, we used barium sulfate and bismuth oxide as main materials for the shielding purpose, which have similar shielding abilities to that of lead, but produce no harm to the human bodies and meet to the purpose of reducing weight.

To make a product that is favorable to wear in the photographing room like an apron, the radiation dose reduction fiber that is suitable for using as a medical purpose is developed in this research. This fiber is made by coating liquid silicon on non-woven fabrics thinly.

In order to verify the usefulness of the radiation dose reduction fiber, we have planned to perform an experiment with several kinds of manufactured radiation dose reduction fibers suitable for different ranges of frequencies of measured free space scattered dose in the radiography room. Hence, free space scattered dose and the rate of shielding, measured from several different distances, are compared with other data in order to estimate the efficiency of shielding for the fibers in the radiography room. On the basis of the results of this experiment, we will show technical data for the quality of the custom-made shielding material appropriate to particular distances from the source and propose the desirable direction for developing technical shielding goods, suitable for a particular environment of works in the future.

## Meterials and Methods

To analyze the effective energy and the capacity of shielding the free space scattered dose of low intensity medical rays for radiation dose reduction fiber, we used the radiography system (Model: UD 150L-40E) made by the Shimadzu company as a device of generating X-rays and used the FH 40 G-L10(2013) of the Thermo SCIENTIFIC company as a digital surveymeter for detecting the free space scattered dose. In addition, we have also used the Exposure and Exposure rate meter(192X, Capintec) and Ion Chamber(Model PM-30, PR-18) in this experiment.

It is well known that the energy represented as a unification for a range of continuous spectrum of X-rays is called effective energy. The half-valued length (HVL) associated with this energy is the same as that obtained from continuous X-rays. We can determine effective energy from the linear attenuation coefficient by using the Hubbell coefficient which is the definition of mass absorption coefficient for a photon with a unificatioin energy^11^. To do this, it is necessary to select the absorbing materials(Al, Cu, Sn, Pb) that will be used for measuring the HVL and to evaluate the linear attenuation coefficient (μ) according the relation


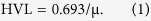


**Figure 1:**
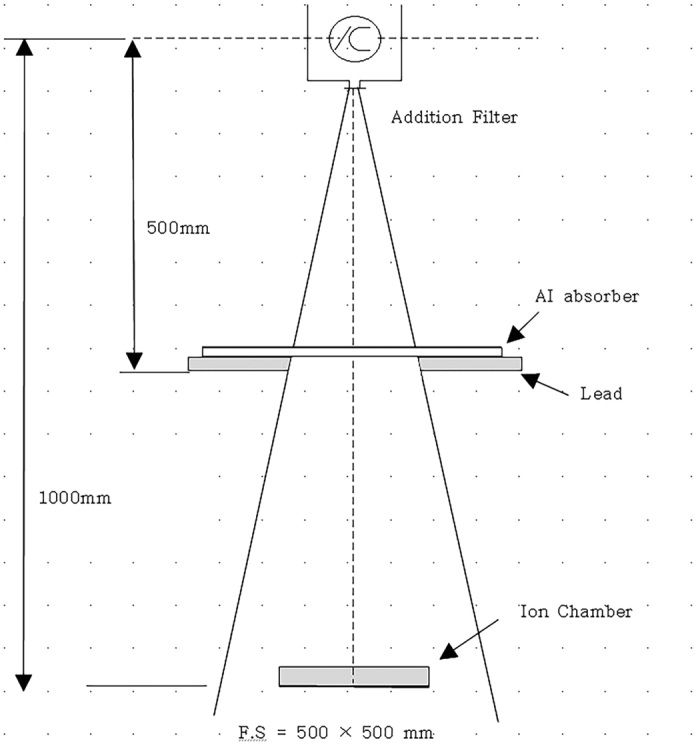
Arrangement for measuring the half value layer.

We have arranged the equipment as shown in Fig. 1 regarding the geometrical condition for the measurement of the effective energy, and, here, have fixed the tube current to be 200 mA, shooting time 0.1 sec, and the inherent filtration 0.7 mmAl. Used tube voltages are 60 kVp(without added filter) and 100 kVp(added filter 0.2 mmCu is attached) which belong to the typically used range. The HVL is obtained under this situation by measuring the exposure dose using the examined and regulated ionizing chamber while we have varied the thickness of the Al absorbing object specially made for measuring the HVL. In order to obtain more exact data for the shielding capacity, we have checked not only the accuracy of the tube voltage and the shooting time, but also the reproducibility and linearity of the power of X-rays before the main experiment.

**Figure 2:**
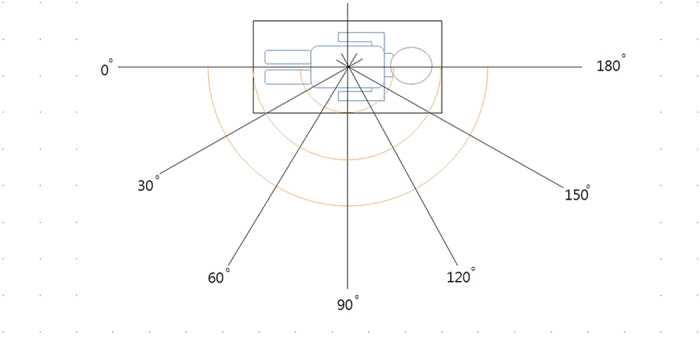
Schematic diagram for the experimental method.

In Fig. 2, we have taken the vertical reference point of the dosimetry locations in the interior of the radiography room on the genital area of a man standing on the floor from which its height from the floor is about 100 cm. From this point in the radiography table, equally spaced seven angles (each angle interval being 30°) of direction are chosen as the location of detection by tracing a semi-circular arc along the horizontal angle as shown in Fig. 2. We have chosen four points for each angle as the spots of measurement, where they are apart 50 cm, 100 cm, 150 cm, and 200 cm from the reference point in the radiography table. Hence, the total number of spots where we measure the data is 28.

Recall that the purpose of this experiment is to evaluate the capacity of the dose reduction fiber, manufactured for the purpose of preventing the human body from absorbing a low dose of radiation considering the situation of frequent exposure in radiation for radiologic technologists and regular visitors. Most of the space scattered doses generated in the radiography room are scattered rays. They are soft rays and the transmission power of these low doses are typically weak. These rays are liable to be absorbed in the air and nearly disappears in the spot apart more than a certain distance from the source. In general, the intensity of the scattering rays is high when the tube voltage is high, when the thickness of subjects reflecting rays is large, and when the field of radiation is large as well. According to this, this experiment for measuring a free space scattered dose is performed after laying down the Whole Body Phantom (PBU-60, Kyoto Kagaku company) on a radiography table under the conditions that the tube voltage is 100 kVp, the tube current 200 mA, and the radiography time 0.1 sec. The shielding rate is analyzed on the basis of the data for the free space scattered dose measured 10 times by adjusting the maximum field angle for probing to be 36 cm × 43 cm.

The results of this experiment is not concerned with the shielding of direct rays but of scattering rays. In order to compare the shielding effects of the shielding sheet on direct rays with that of lead aprons, the shielding capacity of this sheet is meassured with the Ion Chamber (Model PM-30, PR-18) under the condition that keeping the tube voltage 100 kVp and the effective energy 45.61 keV for the case without the added filter 0.2 mmCu.

## Results

### Attanuation of the ray

When a medical ray transmits through a shielding material, its amount and the corresponding energy reduce on account of its interaction with the principal materials of shielding. There is a method for measuring such radiation, which enables us to estimate the original energy loss. We can apply it to the measurement of intensities of the radiation before and after its transmission, which are expected to be different according to the distance from the source. In our research, we will measure energy loss taking place in the photography room. We can also consider to evaluate a shielding rate by restoring the original intensity of energy to an area because we are interested in scattering rays, i.e., the low intensity part of the ray instead of a part of the direct X-ray.

**Figure 3:**
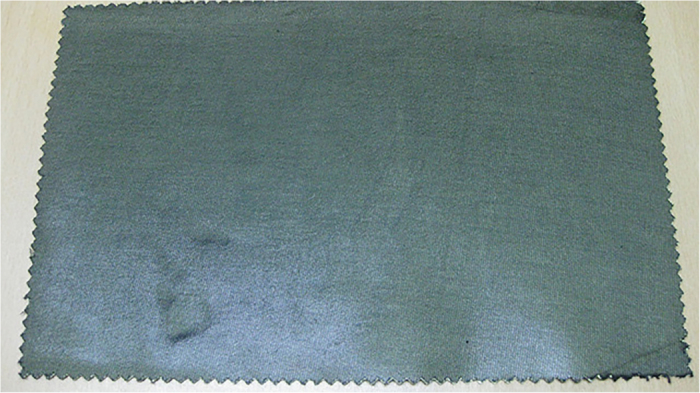
The manufactured radiation dose reduction fiber (Medical radiation dose reduction fiber).

**Figure 4:**
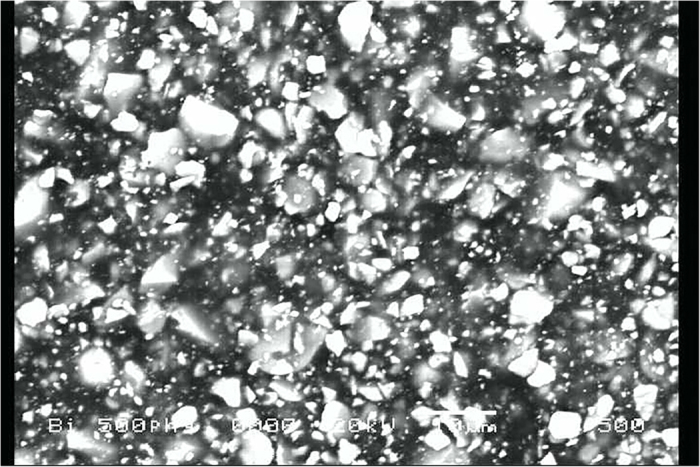
Electron micrograph of the radiation dose reduction fiber. It contains the compound of bismuth and polyolefin resin combinations.

The effects of shielding medical rays using a manufactured dose reduction fiber composed of multi-layers can be represented in terms of intensity loss, which is represented in the form


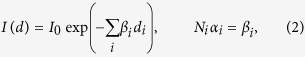


where 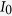 is the intensity of the ray before transmitting the dose reduction fiber, *d*_*i*_ is the thickness of the *i*th layer of the pad of the dose reduction fiber, *β*_*i*_ means the overall area of the material in the *i*th layer that plays the role of shielding in the dose reduction fiber, *N*_*i*_ is the number density (atoms/mm^3^) of atoms for the *i*th layer of the shielding material, and *α*_*i*_ is the average cross section (mm^2^) of the fine absorption of radiation for an atom in the *i*th layer. Notice that 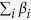 can be explained by the cross section for absorbing low intensity rays that we would like to shield in this experiment, while 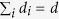 is the total thickness of the pad. To lower the thickness of the pad of the dose reduction fiber and to show the method of enhancing *N*_*i*_*α*_*i*_, we should choose materials composed of high atomic number or should show the method for enhancing *α*_*i*_ which is the microscopic absorption cross-section for radiation in the *i*th layer. In this research, we have chosen a compound of barium sulfate(BaSO4) and bismuth trioxide as a shielding material instead of lead. We have manufactured a thin sheet by combining these materials with the polyethylene resin via forming a pressed compound and we have shown it in Fig. 3 (Fig. 4 is its electron micrograph). In this case, we can achieve the purpose of reducing the weight of the sheet, because it is possible to keep the thickness of the shielding sheet below 1 mm.

In the course of manufacturing the radiation dose reduction fiber, we have observed the experimental method of the lead equivalent regulated from the Korean Standards Association as a Korea Industrial Standard for products used for protecting human bodies from X-rays (KS A 4025, Korean Standards Association, 2010). Three kinds of dose reduction fibers are manufactured. The size of them are 1 m × 1 m and their thicknesses are 0.15 mm (0.039 mmPb), 0.21 mm (0.095 mmPb), and 0.29 mm (0.22 mmPb). The detailed method of the experiment is given in the previous section and illustrated in Fig. 2.

### The measurement of effective energy

The effective energy of X-rays used for measuring the shielding ratio of low dose fiber for medical rays is represented in Table 1. In case of a tube voltage 60 kVp without an added filter, the measured linear attenuation coefficient and the HVL are 0.2886/mm and 2.43 mmAl, respectively. Using the Hubbell’s table for the mass absorption coefficient, we have confirmed that these values correspond to 30.42 keV of effective energy. On the other hand, for the case of the tube voltage 100 kVp with the attachment of an added filter 0.2 mmCu, the measured linear attenuation coefficient and the HVL are 0.1205/mm and 5.62 mmAl respectively, leading to confirming that the corresponding effective energy is 45.61 keV. Hence, we can confirm that the increase of the effective energy and the thickening of the added filter affect the quantity of rays and this leads to the growth of the effective energy. We have also been able to obtain the shielding capacity of low dose fibers from the use of rays that their quality is exactly known.

### The analysis of the free space scattered dose in the area shielded by using radiation dose reduction fibers

Table 2 is shows the dependence of shielding effects on thickness of the shielding material at each point in the photography room measured using a radiation dose reduction fiber that corresponds to 1m^2^. At first, we can confirm from the data associated with no shielding that the distribution of the free space scattered dose in the radiography room decreases according to the distance inverse square law within the photographic condition in the experiment and there are no specific characteristics in the angular distribution of rays.

By using the radiation dose reduction fiber with the thickness of 0.29 mm, that corresponds to the lead equivalent 0.21 mm, we have confirmed that there is an average of 95% shielding effect for each distance from the X-ray source. The radiation dose reduction fiber with the thickness of 0.21 mm, that corresponds to the lead equivalent 0.095 mm, we have confirmed that there is an average of 80% shielding effect for each distance from the X-ray source. The radiation dose reduction fiber with the thickness of 0.15 mm, that corresponds to the lead equivalent 0.039 mm, there is an average of 70% shielding effect for each distance from the X-ray source.

### Analysis of the shielding capacity of the medical radiation dose reduction fiber

As shown in Table 3, the shielding ratio of free space scattering X-rays for the radiation dose reduction fiber is well represented according to the distance from the X-ray source. We had no particular difficulty in manufacturing the dose reduction fiber, because barium sulfate used in this experiment revealed a shielding capacity that is nearly similar to that of lead and, in addition, the flexibility of the materials was satisfactory. We have obtained efficiency results at the spot around 1.5 m from the source, which are nearly identical to that of the existing aprons. If we make an apron that is able to cover from the neck to knee using the radiation dose reduction fibers with the thickness of 0.29 mm, its weight is about 2.75 kg. If we consider that, in general, the operating room and its front door within a hospital, for instance, would exist within 2 m from the general radiography room, it is possible to design the apron with the purpose of its lightweighness. The results of the comparison of our shielding sheets with the existing aprons on shielding effects are given in Table 4. The shielding effect of our sheet exhibits the 75% effect for radiation protection.

## Discussion

The removal of direct X-rays is most important when we shield medical rays that correspond to the range of a diagnosis in medical imaging. The standard capacity of an apron designed for defending medically used radiations is defined in the Korea Industrial Standard (KS P 6023, Korean Standards Association, 2007). According to this definition, the lead equivalent of shielding sheets should be 0.25 mmPb in general, but, for the materials that were used as a shielding partition, the lead equivalent should be more than 0.3 mmPb according to the Korea Industrial Standard (KS P 6024, Korean Standards Association, 2007). Hence, the purpose of the present work is to reduce the weight of the shielding objects using unharmful materials, such as tungsten trioxide (WO_3_), bismuth trioxide (Bi_2_O_3_), and barium sulfate (BaSO_4_), which may be able to be used to replace lead that is commonly used in previous aprons. However, their commercialization is still difficult because there appears more or less serious economic complications when we produce shielding sheets that conform the criteria of both the lightening and the shielding prescription. According to this, we have tried, in this research, the lightening of shielding sheets via the manufacturing of three kinds of sheets that yield their thickness within 0.15 mm~0.3 mm using the compound of barium sulfate (BaSO_4_) and bismuth trioxide (Bi_2_O_3_). This research is initiated with the intention to develop shielding sheets for low dose rays at the level which biological effects are not fully verified, instead of that shielding direct rays, where such low dose rays can be obtained by weakening direct rays. However, because the intensity of rays measured from each distance was reduced than that we had expected, we have resolved the problem of making the sheet weigh lighter by creating shielding sheets that are able to shield low dose rays. Using this, we have proposed a shielding system for medical rays that can be easily used at the level associated with the generation of such medical rays.

However, we have proposed to make a dose reduction fiber which have reduced weight, that can be used to deflect radiation in the range of low intensity as well as high intensity. If we think the fact that the distribution of the free space scattered ray inside radiography room in the medical imaging department is used as an index for the degree of exposure of radiation for the workers and patients, the shielding of radiation is very important for regular employees in radiography rooms^12^,^13^,^14^,^15^. However, it could be inconvenient to wear a massive apron for a worker who is exposed in the range of the free space scattered ray that is quite different from the direct X-ray.

In case that we produce the same apron using the reduced dose fibers with a thickness of 0.15 mm which was suggested in this experiment, we can propose various types of working suits because it is now possible to reduce the weight to be 0.85 kg. In addition, we can propose the same effect of shielding as that of 0.25 mmPb at the distance of 1.5 m from the source. In general, a radiology technologist protects his/her body by attempting to produce radiation behind the defense wall and keeping at least 2 m away even in the case they perform mobile radiography. Hence, we can deduce the result that 0.15 mm reduced dose fiber is enough for defending the radiation shooting.

In situations where work is performed in operating rooms and interventional surgery rooms, employees work for long periods of time wearing an apron. In this case, we can assess, through this research, whether the lightweight textures for low dose rays are more convenient than the existing aprons of which their weights are usually more than 4 kg, for an assistant standing more than 1 meter away from the X-ray source. If we regard that the generation of X-rays for industrial use is typically carried out with an appropriate dose which has pre-defined energy, it would then be possible to adopt an effective method for defending radiologic energy with an expected free space scattered dose in the future.

We have made dose reduction fibers on the basis of the measured data of the free space scattered dose in a radiography room that uses medical rays and their capacities were analyzed in this research, in order to improve the current inferior situation associated with the understandable unwillingness on part of medical employees wearing a very heavy apron. As a result, we measured 0.185 ± 0.009 μSv of the space ray at the spot of 1 m from the 0.29 mm dose reduction fiber coated with a compound of barium sulfate and liquid silicon instead of lead. This means that the dose reduction fiber we have considered can be efficiently used for shielding low intensity radiation. We also have concluded that the results of this research fit in with the purpose of reducing the weight of the apron to be 2.75 kg, while its size is the same as that of the existing ones.

## Additional Information

**How to cite this article**: Kim, S. C. *et al*. Physical analysis of the shielding capacity for a lightweight apron designed for shielding low intensity scattering X-rays. *Sci. Rep.* **6**, 27721; doi: 10.1038/srep27721 (2016).

## Supplementary Material

Supplementary Information

## Figures and Tables

**Table 1:** Effective energies measured for two different specific tube voltages.

Tube Voltage (kVp)	Inh. filter (mmAl)	Add. filter (mmCu)	Abs. coe.(μ) (mm^−1^)	Half value (mmAl)	Eff. energy (keV)
60	0.7	–	0.2886	2.43	30.42
100	0.7	0.2	0.1205	5.62	45.61

**Table 2:** Mean values (Mean ± SD) of the free space scattered dose, depending on thickness of the fiber, after shielding by the radiation dose reduction fiber (unit:μSv).

Thickness	Distance			
	0.5 m	1.0 m	1.5 m	2.0 m
0.00 mm (No shielding)	3.326 ± 0.174	1.692 ± 0.141	0.986 ± 0.050	0.566 ± 0.040
0.29 mm	0.501 ± 0.018	0.185 ± 0.009	–	–
0.21 mm	0.574 ± 0.013	0.269 ± 0.011	–	–
0.15 mm	0.697 ± 0.051	0.320 ± 0.022	0.045 ± 0.008	–

For detailed experimental data with different angles concerning these mean values, you can refer to Supplementary Information.

**Table 3:** Shielding ratio of the free space scattered dose after shielding by the radiation dose reduction fiber (unit: %).

Thickness	Distance			
	0.5 m	1.0 m	1.5 m	2.0 m
0.29 mm	84.88	89.01	100	100
0.21 mm	82.71	84.05	100	100
0.15 mm	79.04	81.05	95.41	99.97

**Table 4:** Results of the comparing of experimental shielding effects of the manufactured sheet with those of existing lead aprons.

Material	Thickness (mm)	Exposure(mR)				Shield ratio(%)
		1	2	3	mean	
Nothing	–	3.40	3.29	3.45	3.38	–
Fiber 1	0.15	2.52	2.61	2.58	2.57	23.9
Fiber 2	0.21	1.71	1.66	1.80	1.75	49.2
Fiber 3	0.29	0.78	0.82	0.81	0.81	75.9
Lead	0.25	0.12	0.15	0.20	0.16	95.3
