# Supplementary material for: Physical analysis of the shielding capacity for a lightweight apron designed for shielding low intensity scattering X-rays
Source: Sci Rep. 2016 Jul 27;6:27721. doi: 10.1038/srep27721 (PMC4962047; doi:10.1038/srep27721)
Supplement: Supplementary Information [file srep27721-s1.doc]

**Physical analysis of the shielding capacity for a lightweight apron designed for shielding low intensity scattering X-rays**

Seon Chil Kim^1^, Jeong Ryeol Choi^2,*^, and Byeong Kyou Jeon^2^

^1^*Department of Biomedical Engineering, School of Medicine,Keimyung University, Dalgubeol-daero 1095, Daegu 42601, Republic of Korea*

^2^*Department of Radiologic Technology, Daegu Health College, Yeongsong-ro 15, Buk-gu, Daegu 41453, Republic of Korea*

^*^Corresponding author, E-mail: choiardor@hanmail.net

**Supplementary Information**

Experimental data for shielding effects of the radiation dose reduction fiber with different angles are represented in this Supplementary Information.

**Table S1**. Free space scattered dose in the radiography room (unit: μSv)

| Distance  Angle | 0.5m | 1.0m | 1.5m | 2.0m |
| --- | --- | --- | --- | --- |
| 0° | 3.540 | 1.630 | 0.974 | 0.638 |
| 30° | 3.410 | 1.700 | 0.952 | 0.548 |
| 60° | 3.440 | 1.880 | 1.030 | 0.526 |
| 90° | 3.450 | 1.876 | 1.070 | 0.604 |
| 120° | 3.170 | 1.670 | 0.997 | 0.548 |
| 150° | 3.180 | 1.580 | 0.949 | 0.536 |
| 180° | 3.090 | 1.510 | 0.929 | 0.560 |
| Mean±SD | 3.326±0.174 | 1.692±0.141 | 0.986±0.050 | 0.566±0.040 |

**Table S2**. Free space scattered dose after shielding by the radiation dose reduction fiber which has 0.29㎜ thickness (unit: μSv)

| Distance  Angle | 0.5m | 1.0m | 1.5m | 2.0m |
| --- | --- | --- | --- | --- |
| 0° | 0.489 | 0.198 | 0 | 0 |
| 30° | 0.502 | 0.182 | 0 | 0 |
| 60° | 0.470 | 0.190 | 0 | 0 |
| 90° | 0.517 | 0.192 | 0 | 0 |
| 120° | 0.498 | 0.169 | 0 | 0 |
| 150° | 0.513 | 0.180 | 0 | 0 |
| 180° | 0.520 | 0.185 | 0 | 0 |
| Mean±SD | 0.501±0.018 | 0.185±0.009 | - | - |

**Table S3**. Free space scattered dose after shielding by the radiation dose reduction fiber which has 0.21㎜ thickness (unit: μSv)

| Distance  Angle | 0.5m | 1.0m | 1.5m | 2.0m |
| --- | --- | --- | --- | --- |
| 0° | 0.595 | 0.259 | 0 | 0 |
| 30° | 0.553 | 0.259 | 0 | 0 |
| 60° | 0.571 | 0.281 | 0 | 0 |
| 90° | 0.578 | 0.282 | 0 | 0 |
| 120° | 0.580 | 0.278 | 0 | 0 |
| 150° | 0.572 | 0.264 | 0 | 0 |
| 180° | 0.568 | 0.260 | 0 | 0 |
| Mean±SD | 0.574±0.013 | 0.269±0.011 | - | - |

**Table S4**. Free space scattered dose after shielding by the radiation dose reduction fiber which has 0.15㎜ thickness (unit: μSv)

| Distance  Angle | 0.5m | 1.0m | 1.5m | 2.0m |
| --- | --- | --- | --- | --- |
| 0° | 0.738 | 0.303 | 0.054 | 0 |
| 30° | 0.711 | 0.310 | 0.056 | 0 |
| 60° | 0.739 | 0.323 | 0.042 | 0 |
| 90° | 0.746 | 0.362 | 0.034 | 0 |
| 120° | 0.692 | 0.319 | 0.045 | 0 |
| 150° | 0.621 | 0.293 | 0.039 | 0 |
| 180° | 0.635 | 0.328 | 0.045 | 0 |
| Mean±SD | 0.697±0.051 | 0.320±0.022 | 0.045±0.008 | - |
